# Axonal Degeneration in AD: The Contribution of Aβ and Tau

**DOI:** 10.3389/fnagi.2020.581767

**Published:** 2020-10-15

**Authors:** Natalia Salvadores, Cristian Gerónimo-Olvera, Felipe A. Court

**Affiliations:** ^1^Center for Integrative Biology, Faculty of Sciences, Universidad Mayor, Santiago, Chile; ^2^Fondap Geroscience Center for Brain Health and Metabolism, Santiago, Chile; ^3^Buck Institute for Research on Aging, Novato, CA, United States

**Keywords:** Alzheheimer’s disease, protein misfolding, axonal degeneration, amyloid β, tau, necroaxoptosis

## Abstract

Alzheimer’s disease (AD) represents the most common age-related neurodegenerative disorder, affecting around 35 million people worldwide. Despite enormous efforts dedicated to AD research over decades, there is still no cure for the disease. Misfolding and accumulation of Aβ and tau proteins in the brain constitute a defining signature of AD neuropathology, and mounting evidence has documented a link between aggregation of these proteins and neuronal dysfunction. In this context, progressive axonal degeneration has been associated with early stages of AD and linked to Aβ and tau accumulation. As the axonal degeneration mechanism has been starting to be unveiled, it constitutes a promising target for neuroprotection in AD. A comprehensive understanding of the mechanism of axonal destruction in neurodegenerative conditions is therefore critical for the development of new therapies aimed to prevent axonal loss before irreversible neuronal death occurs in AD. Here, we review current evidence of the involvement of Aβ and tau pathologies in the activation of signaling cascades that can promote axonal demise.

## Introduction

Alzheimer’s disease (AD) is an adult-onset neurodegenerative disorder and the leading cause of dementia in aged people. While most AD cases are sporadic, less than 5% of the cases are caused by mutations in the amyloid precursor protein (APP) gene or presenilin 1 or 2 genes, leading to excessive production and accumulation of amyloid-β peptide (Aβ; Citron et al., 1992; Hendriks et al., 1992; Mullan et al., 1992; Suzuki et al., 1994; Harvey et al., 2003; Goate, 2006). Many risk factors have been associated with the development of sporadic AD, including the apolipoprotein E ɛ4 allele (Agosta et al., 2009), female gender (Koran et al., 2017), cardiovascular disease risk factors (Samieri et al., 2018), traumatic brain injury (TBI; LoBue et al., 2017) and aging, which is the most important one (Oh et al., 2014). Currently, there is no cure for AD and available treatments can only modestly and briefly alleviate symptoms (Alzheimer’s Association, 2020). The progression of AD, from alterations that include only changes in biomarkers but without the involvement of cognitive decline, to changes that indeed translate into cognitive impairment, follows a continuum that comprises three phases: preclinical AD, mild cognitive impairment (MCI), and dementia due to AD. Clinical symptoms of the disease include deficits in short term memory and language difficulties, as well as behavioral symptoms such as personality changes and depression. Progressive cognitive decline characterized by severe memory loss occurs as the disease progresses. Basic vital functions -such as swallowing- are altered at later stages of the disease, leading to death (Lopez and Dekosky, 2008; Alzheimer’s Association, 2020).

Protein misfolding and accumulation are prominent hallmarks of the disease, with Aβ plaques and tau tangles being the neuropathological signature of AD brains (Wang et al., 2016). Numerous studies have demonstrated that these toxic structures do not act independently and that rather, the neurodegenerative process in AD depends on the interaction between both Aβ and tau (Götz et al., 2001; Lewis et al., 2001; Rapoport et al., 2002; King et al., 2006; Roberson et al., 2007; Hurtado et al., 2010; Ittner et al., 2010; Vossel et al., 2010; Nussbaum et al., 2012; Zempel et al., 2013; Wang et al., 2016). Progressive build-up of these abnormal aggregates is associated with synaptic disruption (Shankar et al., 2008; Koffie et al., 2009; Moreno et al., 2011; Zempel et al., 2013; Rajmohan and Reddy, 2017; Pickett et al., 2019) and neuronal loss (Kadowaki et al., 2005; Jawhar et al., 2012; DeVos et al., 2017; Fu et al., 2017), leading to atrophy of specific brain regions (Spires-Jones and Hyman, 2014; Ferreira et al., 2017; Ten Kate et al., 2018).

Remarkably, the evidence indicates that accumulation of Aβ and tau is slow and that initiates more than two decades before clinical symptoms appear (Jack et al., 2009; Braak et al., 2011; Bateman et al., 2012; Villemagne et al., 2013), which has important diagnostic and therapeutic implications. In this context of early pathological changes during AD, axonal degeneration constitutes a common, initial event in several neurodegenerative conditions (Salvadores et al., 2017). Supporting evidence comes from imaging analyses of individuals with MCI -which are subjects at risk of developing AD- showing a significant decrease in white matter volume. Importantly, these results suggest that atrophy due to disruption of white matter fibers might contribute to memory decline (Kalus et al., 2006; Stoub et al., 2006; Rogalski et al., 2009; Ihara et al., 2010; Bozzali et al., 2011). Additionally, the use of diffusion tensor imaging to examine the microstructural integrity of white matter has revealed a pattern of alterations characteristically observed in axon-related pathologies. These changes correlate with cognitive impairment and are consistent with loss of brain connectivity (Huang and Auchus, 2007; Power et al., 2019).

Acute axonal degeneration, as the result of a traumatic lesion in the central nervous system, is a rapid process that takes hours to days depending on the organism and degenerative stimuli (Court and Coleman, 2012). However, in the case of neurodegenerative diseases, the degeneration of axons might take much longer periods (Lingor et al., 2012). Indeed, histopathological analyses using a mouse model of AD showed that, despite extreme dystrophy, axons maintain continuity throughout the disease course for several months (Adalbert et al., 2009). Moreover, computer modeling using data on neuron loss and neurofibrillary tangle (NFT) formation on AD brains, revealed that NFT bearing neurons can survive for up to 20 years (Morsch et al., 1999). This evidence suggests that neurodegeneration is a slow process in AD, thus providing the opportunity to target degenerating axons as an early therapeutic intervention. Compelling experimental and pathological studies have demonstrated that neurons in AD follow a dying-back pattern of neurodegeneration, where axonal terminals and then axons progressively degenerate toward the neuronal cell body (Bell and Claudio Cuello, 2006; Kalus et al., 2006; Stoub et al., 2006; Huang and Auchus, 2007; Adalbert et al., 2009; Rogalski et al., 2009; Gilley et al., 2011; Nishioka et al., 2019). Despite the difference in timing, axonal degeneration in the context of both acute lesions and neurodegenerative diseases, share morphologic features that include axonal swelling, microtubule disruption, and fragmentation of neuronal processes (Wang et al., 2012), suggesting that they correspond to similar processes. Recent studies have shed light into the mechanisms that govern acute axonal degeneration, revealing the dependence of NAD^+^ in this process, as well as the involvement of mitochondrial dysfunction and necroptosis activation (Barrientos et al., 2011; Osterloh et al., 2012; Neukomm et al., 2017; Hernández et al., 2018; Arrázola et al., 2019; Ko et al., 2020; Loreto et al., 2020; Oñate et al., 2020). Notably, all these pathways are associated to the hallmarks of aging (Kennedy et al., 2014; Sun et al., 2016; Deepa et al., 2018; Haas, 2019; Lautrup et al., 2019; Royce et al., 2019; McReynolds et al., 2020). In the context of AD, different pathogenic pathways have been shown to contribute to axon demise, including calcium signaling imbalance, mitochondrial dysfunction, alterations in axonal transport, and increased oxidative stress (Bamburg and Bloom, 2009; Yu et al., 2009; Ye et al., 2012; Cioffi et al., 2019; Guo et al., 2020). As stated above, the accumulation of misfolded proteins constitutes a salient feature of AD neuropathology and a large body of evidence linking Aβ and tau pathologies with the disruption of axons has been published. In this review, we will present the pathways that contribute to the mechanism of acute axonal degeneration. Then, we will critically evaluate the evidence associating Aβ and tau pathologies with the disruption of axons in AD.

## Cellular Mechanisms Associated with Axonal Degeneration

### Wallerian Degeneration

**Figure 1:**
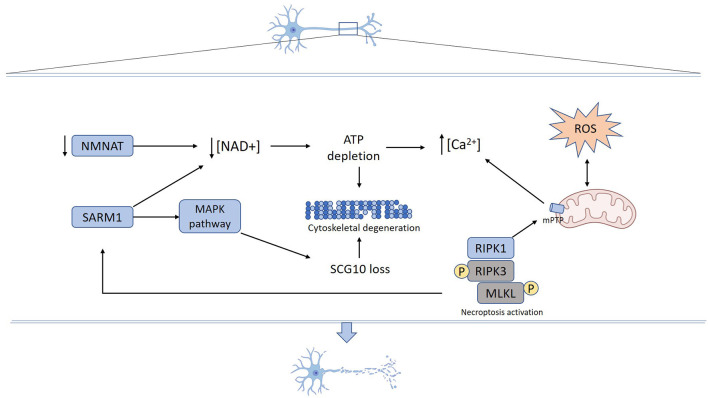
Mechanisms underlying axon degeneration. Mechanical or toxic injury lead to nicotinamide mononucleotide adenylyltransferase (NMNAT) axonal loss and SARM1 activation resulting in NAD+ depletion. Activated SARM1 promotes NAD+ destruction and NMAT loss decreases NAD+ synthesis. Reduced axonal NAD+ levels lead to energy failure and ATP depletion. SARM1 also activates MAPK signaling pathways, which promotes SCG10 proteolysis. Increased reactive oxygen species (ROS) production promotes mPTP formation that also can be triggered by necroptosis activation. Energy failure derived from both NAD+ depletion and mitochondrial damage contributes to calcium unbalance, ROS production, and mPTP formation. Cumulative activation of mechanisms and structural damage ultimately result in cytoskeleton fragmentation and axon degeneration.

The degeneration of axons corresponds to a process activated in response to several stimuli including chemotherapy drugs, infection, inflammation, toxins, and mechanical injury, among others. Recent advances in the study of the molecular mechanisms that govern axon demise have contributed to uncovering essential components of the axon degeneration program. A schematic representation of the steps associated with axonal degeneration is presented in Figure 1.

Initial studies performed by August Waller to study axonal degeneration following nerve transection (Waller, 1850) led to the discovery of an ordered process in which three distinctive phases are typically observed. Initially, a latent period of about 36 h occurs, where the distal injured nerve fiber remains intact. A rapid phase then takes place, where the cytoskeleton is disrupted, and axons undergo fragmentation, which *in vivo* is associated with glial activation (Catenaccio et al., 2017). Finally, axonal disintegration and myelin degradation occurs, followed by macrophage infiltration and clearance of cell debris. This process is known as Wallerian degeneration (Coleman, 2005; Court and Coleman, 2012).

#### NAD^+^ Metabolism in Axonal Degeneration

More than a 100 years later, studies performed on the Wallerian degeneration slow (*Wld^s^*) mutant mice uncovered that upon axotomy, denucleated axons actively execute their own destruction, which is mediated by an evolutionarily conserved signaling pathway (Lunn et al., 1989; Perry et al., 1990, 1991; Lyon et al., 1993). Moreover, the discovery of the *Wld^s^* strain supported the idea that axonal degeneration and cell body death are two events regulated by different molecular mechanisms (Lunn et al., 1989; Deckwerth and Johnson, 1994). In *Wld^s^* mice, the distal denucleated axon remains functional for 3–4 weeks after nerve injury, suggesting that the *Wld^s^* gene confers a protective effect intrinsic to the axon. *Wld^s^* phenotype is caused by the overexpression of a chimeric *Wld^s^* gene, encoding the full-length nicotinamide mononucleotide adenylyltransferase (NMNAT1) and a short region of a ubiquitin assembly protein (UFD2; Conforti et al., 2000). The two components of the *Wld^s^* gene suggest the involvement of the ubiquitin-proteasome system (UPS) and NAD^+^ metabolism in the process of axon degeneration. Although genetic and pharmacological inhibition of UPS activity delays axon degeneration, overexpression of NMNAT1 alone can prevent axonal degeneration (Zhai et al., 2003; Araki et al., 2004). In injured axons, NAD^+^ levels decrease, and preventing this axonal NAD^+^ decline by exogenous application of NAD+ protects axons from degeneration (Wang et al., 2005). Since NAD^+^ is essential for glucose-dependent ATP production, reduced levels of NAD^+^ impair axonal energy production that contributes to axon degeneration (Gerdts et al., 2016). These data suggest that NAD^+^ metabolism plays a crucial role in axon degeneration.

#### SARM1 Mediates Axonal Loss

Mammals harbor three different isoforms of NMNAT proteins (NMNAT 1–3) that differ in subcellular localization and kinetic activity. NMNAT2 is the most labile isoform and it is constantly replenished in axons through fast axonal transport. Upon axotomy, NMNAT2 fails to be transported toward the axons and its levels rapidly drop in axons before Wallerian degeneration occurs (Gilley and Coleman, 2010; Gerdts et al., 2016). Thus, specific depletion of NMNAT2 is sufficient to induce Wallerian-like degeneration of uninjured axons. Moreover, the overexpression of NMNAT3, which is predominantly located in mitochondria, confers axonal protection after injury (Sasaki et al., 2006). The loss of NAD^+^ is suppressed in sterile alpha and TIR motif-containing 1 (SARM1) knockout (ko) axons both *in vitro* and *in vivo*, suggesting that SARM1 is a key mediator of axon destruction (Gerdts et al., 2013, 2015; Gilley et al., 2015). SARM1 contains a C-terminal Toll-interleukin receptor domain, which dimerizes and mediates the rapid breakdown of NAD^+^ (Gerdts et al., 2016). Axonal defects and embryonic lethality observed in *Nmnat2* ko mice are suppressed by *Sarm1* ablation, as *Nmnat2/Sarm1* double-ko mice are healthy. This indicates a relationship between NMNAT2 and SARM1 in the control of NAD^+^ metabolism and axon degeneration. It has been hypothesized that loss of NMNAT2 induces a decline in NAD^+^ levels, which in turn might lead to SARM1-dependent NAD^+^ destruction, and consequently to a disastrous loss of NAD^+^ in the axon (Gerdts et al., 2016).

Although current data indicates that NAD^+^ depletion occurs downstream SARM1 activation after injury, additional evidence suggests the participation of other signaling pathways in axonal degeneration. Forced dimerization of the SARM1 TIR domain which results in NAD^+^ depletion and axon degeneration, also triggers MAPK signaling activation. Moreover, in damaged axons, SARM1 is required for activation of MAPK and this signaling disrupts axonal energy homeostasis leading to ATP depletion (Yang et al., 2015). Thus, the deletion of mitogen-activated protein kinase kinase 12 (MAP3K12), also known as DLK, significantly delays the degeneration of distal axons (Miller et al., 2009). DLK signals through the downstream target of MAPK JNK and pharmacological inhibition of JNK leads to axon protection similar to *Dlk* ablation. Although *Dlk* ablation confers axon protection, this protective effect is weaker than the one resulting from overexpression of *Nmnat* and *Wlds* genes. Also, after injury activation of two additional members of the MAPK cascade has been identified, MEKK4 (MAP3k4) and MLK2 (MAP3K10), which promote axon degeneration. Genetic ablation of all three MAP3Ks leads to robust axonal protection (Yang et al., 2015). All these MAP3Ks converge in the activation of the JNK pathway, which induces UPS-dependent degradation of SCG10 (stathmin 2) after axon injury (Shin et al., 2012).

### The Role of Mitochondria in Axonal Degeneration

As aforementioned, a crucial role of NMNAT in the axonal degeneration cascade has been proven. Supporting studies demonstrated that NMNAT3 overexpression prevents axonal loss mediated by oxidative damage induced by reactive oxygen species (ROS) exposure (Press and Milbrandt, 2008). Additional evidence supported this data and suggested that mitochondrial localization of NMNAT activity has a key role in NMNAT-mediated axonal protection (Yahata et al., 2009). Prompted by this evidence and considering the involvement of mitochondrial permeability transition (mPT) on neurodegenerative conditions (Forte et al., 2007; Du et al., 2008; Martin et al., 2009), Barrientos et al. (2011) sought to determine the role of the mPT pore (mPTP) on the mechanism of axonal degeneration. The researchers showed that degeneration of axons induced by vincristine or nerve transection—in *ex vivo* and *in vitro* models—was associated with activation of the mPTP and targeting the mPTP component Cyclophilin D (CypD), either by pharmacological or genetic means, significantly delayed axonal disintegration (Barrientos et al., 2011). These results identify the mPTP as a key effector of axonal degeneration, as well as a potential target to prevent axonal loss triggered by both mechanical and toxic stimuli. Further studies revealed that upon axonal injury, mPTP formation is mediated by calcium release from the axonal endoplasmic reticulum, constituting an early step in the mechanism of axonal degeneration (Villegas et al., 2014). Moreover, many studies have demonstrated that following axotomy, an increase in intra-axonal Ca^2+^ occurs, constituting a common step to activate the axonal degeneration cascade (George et al., 1995; Adalbert et al., 2012; Avery et al., 2012; Mishra et al., 2013; Vargas et al., 2015). Besides mitochondrial calcium overload, mPTP opening can also be triggered by oxidative stress (Brockemeier et al., 1992). In accordance, *in vivo* work performed in both *C. elegans* and mice, recognized ROS as key intermediates in the mechanism of axonal degeneration, as increasing the anti-oxidative capacity of the neuron efficiently prevented axon demise and functional loss triggered by the hyperactivated degenerin channel MEC-4d (Calixto et al., 2012). Moreover, *in vivo* studies carried out in a mouse model of Charcot-Marie-Tooth 2A disease demonstrated an uncoupling between ATP and ROS production in axonal mitochondria. Similarly, *in vivo*-induced demyelination triggered reduced levels of ATP, along with increased ROS production in axonal mitochondria. These data suggest that mitochondrial ATP and ROS imbalances may contribute to axonal degeneration (van Hameren et al., 2019).

### Necroptosis Involvement in the Mechanism of Axonal Demise

Mitochondrial dysfunction, ROS production, and intracellular calcium increase, which as mentioned above are key events that mediate axonal degeneration, have also been associated with activation of the necroptosis signaling pathway (Vandenabeele et al., 2010). To test the involvement of necroptosis in the mechanism of axonal degeneration, Hernández et al. (2018) used an *in vitro* model of glutamate-induced excitotoxicity in hippocampal neurons seeded in microfluidic devices. The authors showed that axonal degeneration proceeds by necroptosis, which involved the activation of the mPTP as well as calcium dyshomeostasis in axons. Pharmacological inhibition of the necroptotic kinase RIPK1 using Nec-1s, or genetic downregulation of *Ripk3* or *Mlkl*, significantly prevented axonal degeneration and neuronal death (Hernández et al., 2018). In the same line, it was also demonstrated that axonal demise induced by mechanical and toxic stimuli *in vitro*—by axotomy or vincristine, respectively—is dependent on RIPK1, as Nec-1s prevented the degeneration of axons under those conditions. This protective effect was also observed by genetic inhibition of *Ripk*3 o*r Mlkl*. Notably, the loss of the electrophysiological nerve function was also prevented by blocking the necroptotic machinery (Arrázola et al., 2019). Furthermore, investigating the mechanisms of axonal degeneration in *in vitro* and *in vivo* models of Parkinson’s disease, we also uncover the role of the necroptosis signaling pathway in this process. Neurons treated with 6-OHDA exhibited significant axonal degeneration, as well as elevated expression levels of necroptotic markers, which was prevented by Nec-1s or the pMLKL inhibitor GW80. Similarly, intracerebral injection of 6-OHDA in mice triggered axonal degeneration, which was accompanied by RIPK3 and pMLKL upregulation. Nec-1s administration decreased axonal degeneration and improved motor performance of 6-OHDA injected mice, and similar results were obtained by *Ripk3* or *Mlkl* ko (Oñate et al., 2020). Together, these studies confirm that necroptosis activation takes place to mediate the degeneration of axons under different pro-degenerative stimuli, a process we have named necroaxoptosis (Oñate et al., 2020). Recently, a link between necroptosis and SARM1 was discovered using a neuroinflammatory model of glaucoma. TNF-α was injected into the vitreous cavity of wild type mice, triggering axonal and cell body loss, which was prevented in the SARM1 ko mice. Increased levels of necroptosis markers in optic nerves of both wild type and SARM1 ko mice suggested that SARM1 functions downstream of necroptosis mediate axonal degeneration. Additional experiments where necroptosis was induced by direct MLKL dimerization, showed that necroptosis-mediated calcium influx, loss of mitochondrial potential, and axon degeneration were blocked in SARM1 ko axons, integrating previous steps of the axonal degenerative pathway (Press and Milbrandt, 2008; Barrientos et al., 2011; Villegas et al., 2014; Arrázola et al., 2019; Ko et al., 2020). Therefore, necroaxoptosis seems to be a common mechanism for axonal degeneration after a variety of insults.

## The Role of Protein Misfolding in Axonal Degeneration in AD

**Figure 2:**
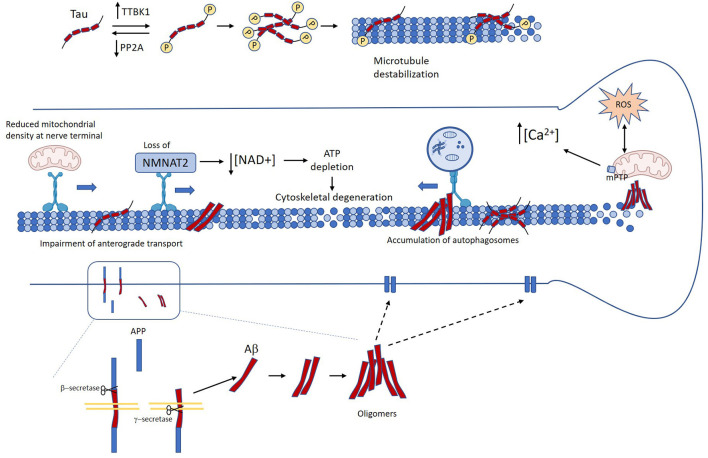
Mechanisms linking Tau and amyloid-β (Aβ) in Alzheimer’s disease (AD) to axon degeneration. Unbalance between kinases and phosphatases leads to the accumulation of abnormally phosphorylated tau, which in turn induces detachment form microtubules and microtubule destabilization (Köpke et al., 1993). Several age-related factors contribute to the accumulation of Aβ oligomers. Accumulation of pathological tau and Aβ promote axonal transport impairment (Calkins and Reddy, 2011; Tang et al., 2012; Wang et al., 2015; Sadleir et al., 2016; Zhang et al., 2018). This in turn causes loss of axonal NMAT2, decreased mitochondrial density at the nerve terminal, and autophagy flux impairment (Gilley and Coleman, 2010; Ljungberg et al., 2011; Ali et al., 2016). Neuron loss and neurofibrillary tangle (NFT) and Aβ oligomers also promote mitochondrial dysfunction, oxidative stress, and calcium dyshomeostasis (Stamer et al., 2002; Chee et al., 2005; Cieri et al., 2018; Mata, 2018; Albensi, 2019). All these events drive the failure of critical mechanisms for axonal functioning and maintenance that lead to loss of axonal homeostasis, and ultimately axon degeneration.

Axonal degeneration and dysfunction are prominent features of AD brains. White matter alterations revealed by *in vivo* imaging can be observed at the early stages of the disease, even in MCI patients. Importantly, these changes correlate with clinical measures of cognitive disability (Kalus et al., 2006; Stoub et al., 2006; Rogalski et al., 2009; Ihara et al., 2010; Bozzali et al., 2011). Accumulation of misfolded proteins is also an early pathological signature during AD, and cumulative evidence documenting a link between these two neuropathological events has been published, which is the focus of this section. A schematic representation of the signaling cascades triggered by Aβ and tau that contribute to axonal degeneration is presented in Figure 2.

### Axonal Disruption Associated With Tau Pathology

The first report describing tau pathology showed that neurofibrillary changes in the form of NFT and neuropil threads (NT) exhibit a characteristic distribution pattern affecting vulnerable brain regions such as the cerebral cortex and hippocampus (Braak and Braak, 1997). These lesions begin with misfolded phospho-tau in the proximal axon and then spreads into the somatodendritic compartment (Braak and Del Tredici, 2011). Deterioration of the cytoskeleton in individual neurons reveals a sequence of changes occurring in neuronal processes suggesting that the disruption of microtubules containing tau may cause the degeneration of axons (Kowall and Kosik, 1987; Braak et al., 1994; Braak and Del Tredici, 2011). Moreover, analysis of hippocampal regions showed that tau inclusions within dystrophic neurites correlate with several measures of the mini-mental state examination, suggesting that these pathological lesions contribute to cognitive dysfunction (Ghoshal et al., 2002).

#### The Role of Tau-Mediated Axonal Transport Disruption in Axonal Degeneration

Due to their polarized nature, neurons rely on an efficient axonal transport system for delivering proteins, lipids, and organelles from the cell body to the axon and synapses. The proper function of axonal transport depends on the correct assembly and functioning of all components including microtubules and motor proteins. Therefore, alterations in axonal transport render neurons vulnerable to the loss of synapses and can trigger axonal degeneration (Mandelkow et al., 2003). Indeed, chemical interventions that directly or indirectly affect axonal transport result in a dying-back form of axonal degeneration (Fukuda et al., 2017). Disruption of axonal transport as well as morphological alterations of the axons occur early in the course of AD and can be detected even a year before other neuropathological abnormalities develop, including amyloid deposition (Stokin, 2005). Defects in axonal transport have been extensively studied in the context of AD and it has been suggested to have a causative role in the disease (Muresan and Muresan, 2009; Vicario-Orri et al., 2014).

In AD, pathological changes associated with tau begin as the granular accumulation of phosphorylated tau in the cytoplasm, axon, and dendrites. Then, tau gradually aggregates to form NT and NFT, which affect the neuronal process that eventually undergoes degeneration (Braak et al., 1994; Braak and Del Tredici, 2011). Tau is a microtubule-associated protein involved in microtubules dynamics, which function as a track for axonal transport (Gao et al., 2018). It has been hypothesized that an imbalance in intracellular signaling causes excessive tau phosphorylation and its subsequent detachment from microtubules. This, in turn, promotes microtubule destabilization and impairment of axonal transport. Several studies have shown that tau overexpression in neurons increases tau phosphorylation and inhibits axonal transport (Stamer et al., 2002; Mandelkow et al., 2003; Chee et al., 2005; Thies and Mandelkow, 2007). As a result of tau-dependent axonal transport inhibition, organelles and vesicles are reduced in cell processes, which lead to energy depletion, decreased oxidative defense, and dying-back of neurites (Stamer et al., 2002; Mandelkow et al., 2003). The NAD^+^ biosynthetic enzyme NMNAT2, a well-known pro-survival factor that inhibits axonal degeneration after injury, depends on constant replenishment by anterograde axonal transport (Gilley and Coleman, 2010). Interestingly, in human AD brains as well as in rTg4510 mice, NMNAT2 expression is reduced, suggesting a possible mechanism by which tau pathology can contribute to axonal degeneration (Ljungberg et al., 2011; Ali et al., 2016). Thus, disruption of axonal transport-associated to abnormal tau phosphorylation might lead to decreased axonal NMNAT2 levels. The unbalance of the NAD^+^/NMN index due to loss of NMNAT2 could lead to SARM1-dependent NAD^+^ destruction in the axon and ultimately axon degeneration. However, this remains to be established.

Also, to regulate microtubule dynamics, tau modulates the function of the motor proteins dynein and kinesin, modifying the axonal transport of proteins and organelles. Enhanced tau phosphorylation also causes synaptic dysfunction as a consequence of energy depletion, associated with a reduced number of mitochondria in the pre-synapsis (Chee et al., 2005). This reduced density of mitochondria in axons and presynaptic terminals directly affects the energy status and calcium buffering. Additionally, it has been shown that tau may directly affect the endoplasmic reticulum-mitochondria interactions and therefore calcium handling (Cieri et al., 2018). Calcium unbalance may lead to activation of calcium-dependent proteases, calpains, which are implicated in the granular disintegration of the axonal cytoskeleton, a hallmark of Wallerian degeneration. Indeed, calpains are abnormally activated in AD brains, and they have been implicated in the development of tau pathology (Mahaman et al., 2019).

Phosphorylation plays a critical role in the regulation of tau functions, including the regulation of microtubule stabilization and assembly. Phosphorylation of tau is increased in AD brains, suggesting an unbalance in tau-associated kinases and phosphatases (Köpke et al., 1993). The expression of tau-tubulin kinase1 (TTBK1), a brain-specific tau kinase, is significantly up-regulated in the frontal neocortical region of the AD brain and colocalizes with NFT-positive neurons (Sato et al., 2006, 2008). Additionally, transgenic mice expressing human TTBK1 show increased tau phosphorylation and significant axonal degeneration in the entorhinal cortex (Sato et al., 2008; Ikezu et al., 2020). On the other hand, the activity of PP2A, the main tau phosphatase, is reduced in AD brains, and the inhibition of PP2A activity in mice models of AD result in tau pathology and cognitive impairment (Wang et al., 2010; Louis et al., 2011; Braithwaite et al., 2012). Strikingly, PP2A activity can be regulated by NMNAT2, hence reduced NMNAT2 expression observed in AD might down-regulate PP2A activity resulting in tau hyperphosphorylation (Cheng et al., 2013). Thus, in AD pathogenesis the unbalance between kinases and phosphatases may lead to abnormally hyperphosphorylated tau, which disrupts axonal transport, triggering axonal degeneration.

Axons depend on the constant remodeling of damaged proteins and organelles to maintain their correct functioning and connectivity (Maday and Holzbaur, 2016). This remodeling depends on homeostatic/degradation systems such as the UPS and autophagy (Korhonen and Lindholm, 2004; Kulkarni and Maday, 2018). Acetylation of tau inhibits the degradation of phosphorylated tau by the UPS, and the accumulation of acetylated tau has been identified in AD brains (Min et al., 2010, 2015). Moreover, the loss of Sirtuin-1, which deacetylate tau, is closely associated with the accumulation of tau in the cerebellar cortex (Julien et al., 2009). Although the UPS is responsible for the degradation of up to 80–90% of proteins including tau, misfolded proteins and aggregates are too large to be processed through the proteasome barrel and impede UPS function by physical occlusion (David et al., 2002; Chung et al., 2019). However, *in vitro* studies have demonstrated that tau aggregates can be degraded by autophagy. Ultrastructural analysis of post-mortem brain samples from AD patients showed the accumulation of autophagic vesicles within swollen and dystrophic neurites. Interestingly, autophagic vesicles were found more frequently in neurons bearing NFT (Nixon et al., 2005). Inducing autophagy by pharmacological interventions in different models of AD results in lower tau accumulation and better cognitive performance (Li et al., 2017). These observations suggest that autophagy impairment is associated with the deposition of pathological tau and contributes to neuronal demise in AD. Transport of autophagic vesicles loaded with unfolded proteins and organelles from distal axonal domains relies on a well-functioning retrograde transport to reach the soma, where autophagy degradation occurs (Maday and Holzbaur, 2014; Kulkarni and Maday, 2018). Dysregulations of tau observed in AD can impair dynein-retrograde axonal transport (Wang et al., 2015), which may lead to autophagy disruption and accumulation of autophagic vesicles within axons. Ultimately all these events might cause the loss of axonal homeostasis that gradually dye back to the soma.

Oxidative stress is another event associated with axonal degeneration in AD at the early stages of the pathologic process (Alavi Naini and Soussi-Yanicostas, 2015). Increased susceptibility to oxidative stress is linked to tau hyperphosphorylation, which leads to peroxisome depletion in neurites due to the inhibition of microtubule transport (Stamer et al., 2002). On the other hand, oxidative stress leads to increased tau phosphorylation in neuronal cultures and animal models of AD (Melov et al., 2007; Su et al., 2010). Indeed, antioxidant treatment reduces oxidative stress, tau pathology, and improves cognitive performance in 3xTg-AD mice (Clausen et al., 2012). An imbalance between pro-oxidants and antioxidants at the early stages of AD leads to increased oxidative stress, which may promote dysregulation of tau phosphorylation. This in turn might promote disruption of axonal transport that exacerbates oxidative stress and tau phosphorylation. Thus, tau phosphorylation and oxidative stress interplay is a key component of a vicious circle that plays a crucial role in the pathological process of AD. Then, ROS production might trigger mitochondrial dysfunction and necroptosis activation, two well-established mechanisms of axonal degeneration.

Analysis of postmortem brain samples suggests that tau pathology begins before the formation of NFT, as the accumulation of hyperphosphorylated tau is observed in young AD patients (Braak et al., 2011). Several lines of evidence suggest that abnormal phosphorylation of soluble tau causes synapse loss, impaired synaptic function, disrupted axonal transport, and cognitive deficits (DeKosky and Scheff, 1990; Callahan and Coleman, 1995; Mandelkow et al., 2003; Thies and Mandelkow, 2007; Hoover et al., 2010). Therefore, it has been hypothesized that NFT occurs as a protective cellular response, where NFT might scavenge the toxic monomeric or oligomeric tau. However, these tau aggregates might sequester other cell components or even cause an axonal clogging that ultimately leads to axonal degeneration (Mandelkow et al., 2003). Thus, tau pathology in AD likely initiates with a signaling dysregulation leading to hyperphosphorylation of tau, which causes microtubule destabilization and tau mislocalization. These events may drive the failure of critical mechanisms for axonal functioning and maintenance that lead to loss of axonal homeostasis. Finally, phospho-tau assembles into NFT, and the aggregation of these tau oligomers eventually contributes to axonal degeneration.

### Evidence Linking Aβ to Axonal Disruption

#### Aβ-Related Axonal Dystrophy

In the first report describing the neuropathology of the postmortem brain of an AD patient, the appearance of altered neuronal processes (referred to as dystrophic neurites) was described (Alzheimer, 1907; article translated in Stelzmann et al., 1995). Subsequently, numerous studies examining AD brain tissue, as well as transgenic animal models of the disease, have revealed an intimate association between dystrophic neurites (i.e., axons and dendrites) and Aβ deposition (reviewed in Spires and Hyman, 2004; Woodhouse et al., 2005; Bell and Claudio Cuello, 2006; Mokhtar et al., 2013). Dystrophic neurites are defined as abnormal neuronal processes, characterized by the presence of thick, tortuous, as well as swollen segments (Su et al., 1993). The morphology of these irregular neuronal processes, which correspond to regions with cytoskeletal alteration and organelle accumulation, may change as the disease progresses (Vickers et al., 1996; Su et al., 1998; Woodhouse et al., 2009; Sanchez-Varo et al., 2012).

Although most research in AD has focused on the study of the loss of dendrites and synapses associated with Aβ pathology, as there is compelling evidence linking these alterations with cognitive decline (Davies et al., 1987; DeKosky and Scheff, 1990; Terry et al., 1991; Scheff et al., 2006; Bastrikova et al., 2008; Jackson et al., 2019), Aβ-related dystrophic axons are widespread in diseased brains and several studies suggest that they contribute to synaptic damage (Adalbert et al., 2009; Sanchez-Varo et al., 2012).

*In vivo* evidence of the temporal course of Aβ-related axonal pathology underwent by different AD mouse models—using two-photon imaging—has revealed that the environment around plaques is not uniform and that rather, it promotes a continuous remodeling, suggesting that axonal dystrophies associated with Aβ plaques are highly plastic structures, with a morphology that varies over time (Tsai et al., 2004; Blazquez-Llorca et al., 2017). Notably, although a re-growth phenomenon has been observed, were newly formed axonal segments were visualized in dystrophic axons, the elimination rates of neurites near amyloid aggregates are significantly higher than the formation rates, thereby leading to a progressive net loss over time (Tsai et al., 2004). As the formation of dystrophic axons seems to be a very dynamic process, with alterations appearing and disappearing within short periods, it has been suggested that early treatment to prevent amyloid accumulation may induce the recovery of the neuronal networks (Blazquez-Llorca et al., 2017). In agreement, different research groups have demonstrated that Aβ clearance by immunotherapy can attenuate Aβ-related axonal degeneration in AD transgenic models (Lombardo et al., 2003; Brendza et al., 2005; Liu et al., 2011).

#### Axonal Transport Disruption Associated With Aβ

Although there is a general notion that the development of dystrophic axons occurs as a consequence of nearby amyloid deposition, a growing body of evidence supports the converse view of an axonal origin of Aβ substrates for extracellular plaque formation, at sites were dystrophies are formed. Thus, the cause and effect relationship between amyloid plaques and axon damage remains a matter of debate.

Altered axonal transport constitutes a typical feature seen in the brain of TBI patients (who are at high risk of developing AD), as well as in TBI animal models (Choe, 2016). Immunohistochemical assessment of TBI brain samples shows increased levels of intra-axonal APP at swelling sites, and this has been attributed to the neuroprotective functions of this protein, as well as the products of its proteolytic processing (Plummer et al., 2016). However, there is well-documented evidence showing that widespread diffuse Aβ plaque deposition occurs in TBI survivors, and this has also been demonstrated in animal models of TBI (Johnson et al., 2012; Shishido et al., 2016; Edwards et al., 2017; Abu Hamdeh et al., 2018). Importantly, longitudinal studies performed to these individuals have revealed that NFT and amyloid plaque pathologies persist, and are associated with cognitive impairment (Johnson et al., 2012). Moreover, compelling studies have demonstrated that alterations in APP transport can promote the local release of Aβ, which can further impair axonal transport (Rodrigues et al., 2012; Mórotz et al., 2019). Thus, just as alterations in axonal transport can lead to amyloid pathology, there is also extensive evidence that Aβ itself can cause microtubule-based transport defects (Pigino et al., 2009; Zhao et al., 2010; Calkins and Reddy, 2011; Tang et al., 2012; Cruz et al., 2018; Zhang et al., 2018). In this line, and considering the role of microtubules in axonal transport, several studies have focused on the effects of Aβ on the integrity of the microtubule network (Fifre et al., 2006; Gevorkian et al., 2008; Silva et al., 2011; Mota et al., 2012; Pianu et al., 2014; Wang et al., 2018; Gao et al., 2019). For instance, Sadleir et al. (2016) hypothesized that presynaptic dystrophies were triggered by Aβ-mediated microtubule disruption. Using live-cell imaging of primary neurons, the authors observed that exposure to Aβ oligomers causes microtubule depolymerization, neuritic beading, and altered axonal trafficking. These data were validated in brain tissue from an AD mouse model as well as in human AD samples, where dystrophic axons and terminals in the proximity to Aβ deposits, displayed aberrant localization of tubulin, as well as evidence of decreased lysosomal function and autophagic vesicles accumulation, suggesting alterations in the microtubule-based transport. Notably, elevated β-secretase-1 and APP levels were also observed in peri-plaque dystrophies, which caused local Aβ generation that may further exacerbate extracellular amyloid pathology (Sadleir et al., 2016). Together, this evidence points towards a mechanism in which Aβ-mediated axonal defects, and the contribution of axonal dystrophies to Aβ plaque formation, are not isolated processes. Rather, a vicious circle seems to occur, where axons in the vicinity of amyloid deposits undergo alterations that further enhance Aβ accumulation.

As discussed above, defects in axonal transport occur early in the onset of AD. Considering the architecture of axons, transport along this structure is critical to preserve its integrity, as a failure of intra-axonal trafficking can lead to deprivation of cargoes that are essential for axon survival and can also impair the communication with the cell body or other cells (Coleman, 2011; Kanaan et al., 2013). Accordingly, disruptions on this vital cellular process can eventually lead to axonal degeneration (Ferri et al., 2003; Coleman, 2005, 2011; Pigino et al., 2009; Kanaan et al., 2013). In this regard, Aβ-mediated disturbances in microtubule-based cellular transport block the trafficking of vital cargoes to synapses. For example, many works reporting impairment of mitochondrial transport induced by Aβ have been published (Rui et al., 2006; Calkins and Reddy, 2011; Kim et al., 2012; Umeda et al., 2015; Zhang et al., 2018). Mitochondria are required at pre- and postsynaptic terminals for the correct function of neurotransmission, as it has essential roles in ATP production and buffering synaptic calcium (Li et al., 2004; Sheng and Cai, 2012). Thereby, the abnormal function of axonal transport induced by Aβ can block mitochondria trafficking, triggering synaptic alterations, which can initiate retrograde degeneration of axons. However, the exact mechanism that mediates this process remains unclear. As previously mentioned, alterations in NMNAT2 are associated with AD (Ali et al., 2016). Although a direct link between Aβ and NMNAT2 has not been proven, it is likely that, in the course of events leading to the activation of the axon death cascade, tau -which indeed has been related to NMNAT2 downregulation (Ljungberg et al., 2011)- acts downstream Aβ.

#### Aβ and the Mechanism of Axonal Degeneration

As previously stated, white matter alterations, reflecting axonal degeneration and dysfunction, are present not only in AD brains but also in MCI patients. Growing data from *in vivo* imaging studies have documented a relationship between such white matter abnormalities and Aβ deposition (Collins-Praino et al., 2014; Hoy et al., 2017; Schilling et al., 2018; Vipin et al., 2019; Weaver et al., 2019; Caballero et al., 2020). Importantly, recent longitudinal studies on the association between white matter integrity and amyloid pathology in cognitively normal individuals, revealed that Aβ aggregates build-up in white matter fibers known to be affected in AD, in an age-dependent manner (Vipin et al., 2019; Caballero et al., 2020). These studies underscore the impact of Aβ load on early white matter alterations in normal aged subjects at risk of AD. For this reason, a thorough understanding of the signaling that mediates axonal degeneration is crucial, as this would allow the identification of potential therapeutic targets.

Several lines of investigation have demonstrated that rather than senile plaques, Aβ oligomers are the most cytotoxic form of the aggregates and that plaques might serve as reservoirs from which oligomers can diffuse, reaching cellular targets in their vicinity. Aβ oligomers can exert toxicity through several different pathways, and many of them are involved in the process of axonal degeneration, including mitochondrial dysfunction (Swerdlow, 2018; Albensi, 2019), oxidative stress (Butterfield and Boyd-Kimball, 2018; Cheignon et al., 2018) and calcium dyshomeostasis (Mata, 2018; Popugaeva et al., 2018; Wang and Zheng, 2019). As discussed, previous work by our group demonstrated that axonal degeneration triggered by distinct insults is dependent on mitochondrial dysfunction and activation of the mPTP (Barrientos et al., 2011). In this line, Aβ oligomers were shown to target the mPTP regulator cyclophilin D (CypD) in a transgenic mouse model of AD as well as in AD brain samples. In this animal model, CypD deficiency prevented Aβ-induced mitochondrial swelling and permeability transition, improved calcium buffering capacity, and decreased mitochondrial ROS (Du et al., 2008, 2011). *in vitro*, the absence of CypD protected neurons from Aβ- and oxidative stress-mediated cell death. Remarkably, inhibiting the mPTP by genetic ablation of CypD improved cognitive and synaptic function in the transgenic AD model (Du et al., 2008, 2011). Furthermore, Aβ-induced impairment of axonal mitochondrial trafficking depends on CypD-mediated mPTP activation. Genetic deletion of CypD suppressed Aβ-mediated activation of the p38/MAPK signaling pathway, reversed axonal mitochondrial alterations, improved synaptic function, and prevented synapse loss (Guo et al., 2013). These studies shed light on the mechanism by which Aβ triggers mitochondrial dysfunction, which may promote axonal degeneration in AD.

As mentioned, both oxidative stress and calcium dyshomeostasis have shown to mediate axonal degeneration, and these two events have been largely studied in the context of Aβ-mediated neurodegeneration. However, most of the works have focused on the neuron as a whole, instead of studying the subcellular mechanisms that are activated in response to Aβ. Thus, to comprehend the exact pathways that trigger the loss of axonal structures, the use of compartmentalized microfluidic devices, which allow the isolation of axons from cell bodies and dendrites, represent a useful tool. In this regard, Song et al. (2006) used a compartmented chamber to study the differential effects of Aβ in somas and axons of sympathetic neurons. The researchers demonstrated that exposure of axons to Aβ triggered a caspase-independent mechanism of degeneration, which lead to nuclear apoptosis, as caspase inhibitors prevented apoptosis but did not protect neurons from axonal degeneration. Interestingly, the treatment of axons with calpastatin to inhibit calpains, which have been shown to mediate axonal cytoskeleton disintegration during Wallerian degeneration, not only protected axons from degeneration but also prevented nuclear apoptosis (Song et al., 2006). Using the same approach, another research group found that exposure of axons from hippocampal neurons to Aβ oligomers activated intra-axonal translation and induced local ATF4 synthesis. Retrograde transport of the protein to the soma induced ATF4-dependent gene expression and cell death. Similarly, intracerebral injection of Aβ in mice led to ATF4 translation in cholinergic axons, which was required for the retrograde transmission of Aβ-induced neurodegeneration (Baleriola et al., 2014; Walker et al., 2018).

As described here, recent studies have unveiled that axonal degeneration depends on necroaxoptosis activation (Hernández et al., 2018; Arrázola et al., 2019; Ko et al., 2020; Oñate et al., 2020). Although necroaxoptosis has not been demonstrated to occur in the context of AD, the evidence of necroptosis activation in AD brains (Caccamo et al., 2017) suggests that—similar to the findings in PD (Oñate et al., 2020)—this process may indeed contribute to axonal demise in AD.

### Synergistic Contribution of Aβ and Tau to Axonal Degeneration

Mounting data has proven not only a synergistic but also a dependent neurotoxic effect of Aβ and tau pathologies (Bloom, 2014). To *in vivo* assess the pathological mechanism of Aβ and tau interaction on axonal degeneration, Nishioka et al. (2019) used a transgenic tau model and injected Aβ aggregates into the axonal terminals of retinal ganglion cells. Diffusion tensor imaging revealed a progressive white matter loss that correlated with the histopathological observation of retrograde axonal degeneration. Moreover, Aβ exposure triggered tau phosphorylation that preceded axon loss, and treatment with the microtubule-stabilizing compound Epothilone D inhibited tau phosphorylation and prevented axonal degeneration (Nishioka et al., 2019). This work emphasizes the cross-talk between Aβ and tau proteins that trigger the degeneration of axons and represents the first study to *in vivo* demonstrate the influence of this interaction in the mechanism of axonal degeneration. However, the exact signaling pathway activated downstream Aβ and tau interaction, and responsible for the disintegration of the axonal cytoskeleton, has not yet been explored.

Previous works have shown that reductions in tau can prevent Aβ-induced neurodegeneration and cognitive alterations in AD mouse models (Roberson et al., 2007; Ittner et al., 2010). Considering both the evidence of Aβ-mediated axonal transport deficits, as well as the interaction between tau and Aβ to induce neuronal alterations, Vossel et al. (2010) studied the effects of Aβ on axonal transport of mitochondria and TrkA, in hippocampal neurons from tau deficient and wild type mice. The researchers found that Aβ-dependent inhibition of axonal transport of mitochondria and TrkA was prevented in tau^−/−^ neurons, stressing that a dependent degenerative effect between both proteins might take place in AD (Vossel et al., 2010). In line with this, i*n vitro* and *in vivo* experiments showed that Aβ trimers derived from postmortem human AD brains, induced conformational changes in tau that led to reduced expression of the kinesin-1 light chain and increased intra-axonal APP, suggesting disruption of axonal transport (Sherman et al., 2016).

Although axonal transport deficits associated with misfolded amyloid and tau have been largely studied in AD brains and models, the signaling cascades acting downstream these alterations that might activate axonal degeneration pathways are not clearly defined. Thus, the evidence demonstrates that axonal degeneration is an early event in AD, where Aβ and tau pathologies interplay contributes to the degenerative process. Nevertheless, besides the reduced NMNAT2 levels found in AD brains, there is no evidence of the involvement of key molecules that govern Wallerian degeneration—such as necroptosis-associated proteins or SARM1—in the context of AD. Moreover, whereas Wallerian degeneration proceeds rapidly, the evidence indicates that axonal degeneration in AD rather constitutes an extended process that may take months or even years. Hence, it is possible that, although some common players are indeed involved, a mechanism different from the one activated during Wallerian degeneration mediates axonal degeneration in AD.

## Concluding Remarks

Aging is considered the main risk factor contributing to the development of neurodegenerative diseases, including AD. The age-dependent decrease of homeostatic systems such as chaperones, degradation systems, and antioxidant defense considerably contributes to the accumulation of misfolded proteins in the brain. Although deposition of Aβ and tau has been the center of AD drug development research, many clinical trials on Aβ and tau clearing have been conducted, with disappointing outcomes (Congdon and Sigurdsson, 2018; Huang et al., 2020). As discussed in this review, a large body of evidence has uncovered a link between the development of Aβ and tau pathologies and the process of axonal degeneration. Nonetheless, a clear mechanism that integrates the known signaling cascades that activate axonal death, with the pathways that have been shown to mediate axonal degeneration in the context of AD, remains elusive. Many reports have documented that significant decreases in NAD^+^ occur during aging (Lautrup et al., 2019; McReynolds et al., 2020) and supplementation of this metabolite has been suggested as a potential treatment for age-related diseases including AD (Braidy et al., 2018; Hou et al., 2018). Despite the importance of NAD^+^ in the mechanism of axonal degeneration, its role in axon demise in AD is undefined. Furthermore, whether necroaxoptosis is activated in AD, is still unknown. Axonal loss in AD appears to be a slow process that initiates early during disease progression, thus providing a window for therapeutic intervention. An in-depth understanding of the mechanism that governs axonal degeneration is critical for the development of new therapies that allow to halt axonal loss and therefore prevent cell death and cognitive decline in AD.

## Author Contributions

NS planned and structured the manuscript, researched, and wrote the review. CG-O researched and wrote the review. FC revised the manuscript. All authors contributed to the article and approved the submitted version.

## Conflict of Interest

The authors declare that the research was conducted in the absence of any commercial or financial relationships that could be construed as a potential conflict of interest.
